# Increased risk for postoperative periprosthetic fracture in hip fracture patients with the Exeter stem than the anatomic SP2 Lubinus stem

**DOI:** 10.1007/s00068-019-01263-6

**Published:** 2019-11-18

**Authors:** Carl Mellner, Jabbar Mohammed, Magnus Larsson, Sandra Esberg, Maciej Szymanski, Nils Hellström, Cecilia Chang, Hans E. Berg, Olof Sköldenberg, Björn Knutsson, Per Morberg, Sebastian Mukka

**Affiliations:** 1 Department of Surgical and Perioperative Sciences, Umeå University, Umeå, Sweden; 2 Department of Surgical and Perioperative Science (Orthopedics), Sunderby Research Unit, Umeå University, Umeå, Sweden; 3 Department of Clinical Sciences, Intervention and Technology (CLINTEC), Karolinska Institutet, Stockholm, Sweden; 4 Department of Clinical Sciences At Danderyd Hospital (KIDS), Karolinska Institutet, Stockholm, Sweden

**Keywords:** Hip fracture, Periprosthetic fracture, Femoral neck fracture, Hip arthroplasty, Mortality

## Abstract

**Background:**

The purpose of this study was to compare the cumulative incidence of postoperative periprosthetic fracture (PPF) in a cohort of femoral neck fracture (FNF) patients treated with two commonly used cemented stems: either a collarless, polished, tapered Exeter stem or the anatomic Lubinus SP2 stem.

**Methods:**

In this retrospective multicenter cohort study of a consecutive series of patients, we included 2528 patients of age 60 years and above with an FNF who were treated with either hemiarthroplasty or total hip arthroplasty using either a polished tapered Exeter stem or an anatomic Lubinus SP2 stem. The incidence of PPF was assessed at a minimum of 2 years postoperatively.

**Results:**

The incidence of PPF was assessed at a median follow-up of 47 months postoperatively. Thirty nine patients (1.5%) sustained a PPF at a median of 27 months (range 0–96 months) postoperatively. Two of the operatively treated fractures were Vancouver A (5%), 7 were Vancouver B1 (18%), 10 were Vancouver B2 (26%), 7 were Vancouver B3 (18%), and 13 were Vancouver C (32%). The cumulative incidence of PPF was 2.3% in the Exeter group compared with 0.7% in the SP2 group (*p* < 0.001). The HR was 5.4 (95% CI 2.4–12.5, *p* < 0.001), using the SP2 group as the denominator.

**Conclusions:**

The Exeter stem was associated with a higher risk for PPF than the Lubinus SP2 stem. We suggest that the tapered Exeter stem should be used with caution in the treatment of FNF.

**Trial registration:**

The study was registered at clinicaltrials.gov (identifier: NCT03326271).

## Background

Postoperative periprosthetic fracture (PPF) is a rare but increasing and severe complication after hip arthroplasty, especially in elderly and fragile hip fracture patients [1–4]. Extensive revision surgery contributes to a high mortality rate and, in those who prevail, makes recovery difficult [5, 6]. Several risk factors for PPF have been proposed, including advanced age, sex [7, 8], osteoporosis, rheumatoid arthritis [9], and femoral neck fracture (FNF) as an indication for primary surgery [10]. The treatment of PPF can be technically demanding and is plagued with a high frequency of complications due to deep infection, dislocation, and intraoperative fractures [11, 12].

The two most commonly used cemented implants in Sweden for treating a displaced FNF are the polished, tapered Exeter stem and the matte, anatomic Lubinus SP2 stem [13]. Recent reports have identified a high incidence of PPF associated with the Exeter and the similarly tapered (CPT, Zimmer, Inc., Warsaw, IN, USA) stems in elderly FNF patients [10, 14–16]. There are few cohort studies comparing the Exeter and Lubinus femoral stems regarding the risk for PPF in patients with hip fractures.

The purpose of this study was to compare the cumulative incidence of postoperative periprosthetic fracture (PPF) in a cohort of femoral neck fracture (FNF) patients treated with two commonly used cemented stems: either a collarless, polished, tapered Exeter stem or the anatomic Lubinus SP2 stem.

## Methods

### Study setting

This retrospective cohort study was performed between 2006 and 2014 at three Swedish hospitals: the orthopedic department of Sundsvall Hospital, the orthopedic department of Sunderby Hospital, and the orthopedic department of Karolinska University Hospital Huddinge, Stockholm. Sundsvall and Sunderby are emergency hospitals affiliated with Umeå University, while Karolinska University Hospital Huddinge is affiliated with the Karolinska Institutet. The combined catchment area consists of approximately 600,000 inhabitants. The guidelines of the STROBE (STRrengthening the Reporting of OBbservational studies in Epidemiology) statement were followed.

### Participants

We included all patients above 60 years of age who were admitted to the participating hospitals between 2006 and 2014 and underwent primary hip arthroplasty for a displaced FNF with either a cemented Exeter stem or a cemented Lubinus SP2 stem. Patients with pathological fractures were excluded.

### Data collection and follow-up

Using the unique Swedish personal identification number, we collected data retrospectively from a combination of digital medical charts. For Karolinska University Hospital Huddinge and Sundsvall Hospital, we also used the SHAR to search for reoperations performed at other hospitals in Sweden. All hip-related complications in the study were managed and registered in our departments and no other reoperations were found to have occurred at other hospitals in Sweden. All patients were followed until 2017 or until death via medical database searches and the minimum follow-up time was 24 months. We collected patient data, including age fracture, sex and comorbidities registered at primary surgery, the ASA score, the type of arthroplasty (total hip arthroplasty (THA)/hemiarthroplasty (HA)), the surgical approach (direct lateral or posterolateral), all surgically treated PPFs, and those Vancouver B and C fractures treated conservatively.

For PPF patients, the radiographs were analyzed by the authors and graded according to the Vancouver classification [17].

### Implant and surgery

At all three hospitals, cemented HA and THA are the standard treatments for a displaced FNF in patients with a biological age more than 65 years and medically fit for arthroplasty surgery. Thus, a number of patients with a chronological age between 60 and 65 received a hip arthroplasty. At Karolinska University Hospital Huddinge, the Exeter stem (150 mm, Stryker Howmedica, Kalamazoo, MI, USA) was used. At Sundsvall Hospital, the SP2 stem (Waldemar Link, Hamburg, Germany) is the standard treatment. At Sunderby Hospital, both the Exeter and SP2 stems are used according to the surgeons’ preference. The Lubinus SP2 (150 mm) is an anatomic cobalt–chromium stem (Waldemar Link). A modular 28-mm or 32-mm cobalt–chrome femoral head was used for THA and either a unipolar head (Unipolar; Waldemar Link) or a bipolar head (Vario cup; Waldemar Link) was used for HA. The Exeter stem has a double-tapered shape combined with a highly polished surface and collarless design. The surgical approach was either a direct lateral approach or a posterolateral approach, depending on the surgeons’ preference. Antibiotic-loaded bone cement was used for all patients. Prophylactic antibiotics were administered 30 min preoperatively and two more times over 24 h postoperatively. Low molecular weight heparin was administered for 14–30 days postoperatively.

Patients were mobilized according to a standard physiotherapeutic program and full weight bearing with the use of crutches was encouraged. Patients who underwent surgery with a posterolateral approach were instructed to minimize flexion in combination with adduction and internal rotation for the first 3 months. Primary surgery was performed either by a consultant orthopedic surgeon or by a registrar.

### Statistical analysis

The Student’s t-test and Mann–Whitney *U* test were used for continuous normal and ordinal data, respectively. All tests were two sided.

We used a Cox proportional hazards for regression modeling with follow-up time as time to death, PPF, or end of follow-up (min 2 years after surgery). The selection of variables for the analyses was an a priori hypothesis based on the literature search for known predictors of the outcome of interest. Our main outcome variable was the presence of a PPF during the study period and we adjusted for exposure variable (type of stem), age, sex, surgical approach (direct lateral or posterior), and type of arthroplasty (hemi- or total hip arthroplasty) achieving 8–10 events per predictor variable. The assumption of parallel lines for the two groups in the log–log cumulative hazard plot were fulfilled.

Results are presented as hazard ratios (HRs) with 95% confidence intervals (95% CI).

Due to different distributions of stems between the centers, we performed sensitivity analyses; first by adding hospital as a covariate to the Cox proportional hazards analysis; second by only analyzing patients treated at Sunderby hospital where both stems were at use.

The statistical analysis is based on the assumption that the studied observations are independent; therefore, no bilateral fractures were included. In patients with two fractures during the study period, only the first fracture was included. The effect of a larger Exeter stem size was analyzed by logistic regression analysis. Mortality was presented as percentages of patients deceased during the first year postoperatively.

Statistical analysis was performed using SPSS Statistics software version 22.0 for Mac (SPSS, Inc., Chicago, IL).

### Ethics and registration

The study was conducted in accordance with the ethical principles of the Helsinki Declaration and was approved by the regional Ethics Committee in Umeå (entry number 2018-205-32M and 2016-113-31M).

The study was registered at clinicaltrials.gov (identifier: NCT03326271).

## Results

### Study subjects and descriptive data

During the study period, 1326 and 1202 hip arthroplasties were performed with the Exeter and SP2 stems, respectively. One patient was excluded due to insufficient documentation. The mean age in the cohort was 82 years (60–103 years). The baseline characteristics of the study group are presented in Table 1. In the Exeter group, patients were slightly older with a higher ASA category and more frequently treated using the direct lateral surgical approach. The median follow-up time was 47 months (range 0–138 months).

**Table 1:** Characteristics of patients

	Exeter (*n* = 1326)	SP2 (*n* = 1202) sig
Sex^a^		
Male	417 (31%)	379 (32%)
Female	909 (69%)	823 (68%)
Age, years^b^	82 (8)	81 (8)
ASA category^a^		
1–2	416 (32%)	456 (38%)
3–4	905 (68%)	600 (50%)
Missing	5 (0%)	146 (12%)
Hospital		
Huddinge	773 (58%)	0 (0%)
Sunderby	553 (42%)	588 (49%)
Sundsvall	0 (0%)	614 (51%)
Type of arthroplasty^a^		
THA	216 (16%)	208 (17%)
HA	1110 (84%)	994 (83%)
Surgical approach^a^		
Posterolateral	417 (31%)	958 (80%)
Direct lateral	909 (69%)	244 (20%)

Individual patients are presented

^a^*n* (%)

^b^Mean (SD)

### Outcome

During the study period, 39 (1.5%) PPFs were identified at a median time of 27 months (0–96 months) postoperatively. None of these PPFs were intraoperative.

The cumulative incidence of PPF was 2.3% in the Exeter group and 0.7% in the SP2 group (*p* = 0.002); the HR was 5.4 (95% CI 2.4–12.5, *p* < 0.001) using the SP2 group as the denominator. The male sex was also linked to an increased risk (2.5, 1.3–4.7, *p* = 0.005), whereas the type of arthroplasty (HA vs THA**,** 0.7, 0.2–2.0, *p* = 0.7), the surgical approach (lateral vs posterolateral, 1.1, 0.6–2.4, *p* = 0.7), and age (1.04, 1.0–1.1, *p* = 0.11) were not associated with an increased risk for PPF. The sensitivity analyses found similar results. First by adding treated hospital as a covariate to the Cox proportional hazards analysis (HR 4.8, 95% CI 2.1–10.6, *p* < 0.001) and second of by only analyzing patients treated at Sunderby hospital (HR 5.5, 95% CI 1.8–16.3, *p* = 0.002). The overall 1-year mortality rate was 23% (585 of 2528 patients).

### Periprosthetic femur fractures

Two of the treated fractures were Vancouver A (5%), 7 were Vancouver B1 (18%), 10 were Vancouver B2 (26%), 7 were Vancouver B3 (18%), and 13 were Vancouver C (33%) (Table 2). In the Exeter group, two B1 fractures were treated conservatively. In the Lubinus SP2 group, one B1 and three C fractures were treated conservatively.

**Table 2:** Type of periprosthetic fracture, surgical treatment and surgical outcome

Vancouver classification	Exeter	SP2
Vancouver A	2 (0.1%)	0 (0%)
Vancouver B1	6 (0.5%)	1 (0.1%)
Vancouver B2	10(0.8%)	0 (0%)
Vancouver B3	7 (0.5%)	0 (0%)
Vancouver C	5 (0.4%)	8 (0.6)
Total	30 (2.3%)	9 (0.7%)
Conservative treatment	6	
Surgical treatment		
Open reduction and internal fixation	20	
Stem revision	13	
Surgical outcome		
Deep infection reoperation	3	
Failure of osteosynthesis	2	
Refracture	1	

All conservatively treated PPFs needed no further surgery. Six of 33 (18%) surgically treated PPFs needed revision surgery due to deep infection (*n* = 3), failure of osteosynthesis and re-osteosynthesis (*n* = 2), and revision arthroplasty (*n* = 1). Two out of 10 (20%) patients with B2 were reoperated and two of seven B3 (28%) fractures. Both patients with B2 fractures were managed with open reduction and internal fixation and required reoperation due to deep infection and mechanical failure with stem revision. Both patients with B3 fractures were managed with stem revision and underwent reoperation due to deep infection and stem revision.

The 1-year mortality rate after PPF was 31% (12 of 39 patients).

## Discussion

In this retrospective cohort study including a large cohort of elderly patients treated with hip arthroplasty for an FNF, the use of the tapered Exeter stem resulted in a higher incidence of PPF than did the anatomic Lubinus SP2 stem.

PPF is a severe complication and the surgical treatment of PPF is often challenging due to revision surgery and an increased risk for postoperative readmission due to surgical complications [18, 19]. Osteopenia, hip fracture as an indication for primary surgery, age greater than 80 years and the use of polished tapered femoral stems have all been associated with an increased incidence of PPF and a predisposition for Vancouver B2 PPFs [10, 20–22]. In a previous study from our institution, we found that the polished, tapered CPT stem was associated with a high incidence of early PPF in an FNF population more than 80 years of age [15]. In the present study, we investigated the more commonly used Exeter stem in comparison to the anatomic Lubinus SP2 stem.

In concordance with our findings, a number of recent studies has also shown association between cemented, polished, tapered stems and a high risk for PPF [10, 14–16]. A Scandinavian registry-based study found that the cemented Exeter stem was associated with a fivefold increased risk for PPF compared to the SP2 stem [23]. Inngul and Enocson [14] published an incidence of PPF (2.3%) identical to that found in the present study using the Exeter stem. A retrospective study by Raut and Parker [24] reported a PPF incidence of 1.0% for the Exeter stem. In contrast, two recently published studies of Exeter stems reported a higher incidence of PPF for Exeter stems than for stems with a composite beam design [18, 24, 25]. Palan et al. [25] analyzed data from the National Joint Registry (NJR) of England, Wales and Northern Ireland on 257,202 primary THAs with cemented stems and found a higher incidence of revision surgery for a PPF with the use of a CPT stem than with the use of an Exeter stem [24]. However, there are differences in the design features between the polished, tapered CPT and Exeter stems, which might contribute to these differences. The CPT stem is a chrome–cobalt stem and the Exeter stem is a stainless steel construct. The CPT stem has a more rectangular proximal cross-sectional shape with a 12/14 Morse taper and the Exeter stem has a V-40 taper.

A tapered stem designed to subside in the cement mantle may act as a stress riser, creating an axial load and in turn splitting the femur after a hip contusion in a complex Vancouver B PPF. The SP2 stem is designed for a distal femoral neck osteotomy, which visualizes the femoral canal and, together with the anatomically shaped stem design, might facilitate better alignment [26]. The distal anchoring of the stem, the favorable positioning of the SP2 stem, and the avoidance of the stress rising caused by the wedge design of the Exeter stem might be the reasons for the lower risk for PPF in this high-risk population. The effect of collar has been investigated in the literature [27]. In a randomized RSA study of different designs of the Lubinus stems indicated that alternative shapes could be possible and give adequate clinical results [28].

The size of the Exeter stem has been proposed as a risk factor for PPF and choosing smaller stem sizes has been considered to lower the risk for PPF by increasing the cement mantle. A larger stem size could increase the risk for endosteal contact of the tip of the prosthesis by decreasing the cement mantle, which in turn increases the stress rising after a hip contusion [29]. The surgical approach has been proposed to influence the rate of PPF due to an increased risk for anteroposterior malalignment. However, in our previous study, we did not find any statistical association between the direct lateral approach and the risk for PPF [10, 15]. In concordance with Inngul and Enocson [14], we found that the male sex was associated with an increased risk for PPF. There might be differences between the populations studied, because the female sex has been reported to be a risk factor in the osteoarthritic population [7, 14].

The 1-year mortality rate after PPF has been 7–18% [19, 30] compared to that of 8–36%, among hip fracture patients [31]. In the FNF population, several factors affect 1-year mortality such as age, cognitive impairment, and the 1-year mortality rate are in line with previous reports in the literature [32, 33], prefracture mobility, and habitat [3]. In the present study, we found a staggering 1-year mortality rate of 31% after PPF. These results indicate the severity of PPF in this fragile population. Previous studies on the mortality rate of PPF have reported conflicting results in comparing open reduction and internal fixation versus revision THA [18, 34, 35]. Boylan et al. [35] found that revision arthroplasty was associated with a higher risk of short-term mortality up to 6 months, but a similar mortality rate at 1 year when compared to open reduction and internal fixation [35].

The surgical treatment of PPF requires meticulous preoperative planning, surgeons with competence in hip arthroplasty revision and trauma and a center with the equipment for rapid changes in the surgical plan is worthwhile. The management of PPF depends on the location and configuration of the fracture, the stability of the stem and the bone stock, and these management strategies are described by the Vancouver classification [36].

The Exeter stem has proved to be reliable with good long-term results in elective hip surgery [37]. The low numbers of aseptic loosening of tapered stems might decrease the risk for late periprosthetic fractures. We suggest that in this elderly population at risk for PPF, a cemented anatomic stem would decrease the risk for revision surgery [10, 14, 15]. Future research would further identify subgroups of patients that would benefit from different stem designs.

The strengths of our study include the consecutive series of patients who underwent primary arthroplasty for FNF and the follow-up based on the Swedish personal identification number. The main limitations of the study include its retrospective design and the collection of data from different hospitals with either selective use of one or the use of both types of stems. This lead to inherited confounders and biases that are difficult to address. However, we sought to address these issues in the regression analysis. Using the unique Swedish personal ID number, we collected data retrospectively by searching of our in-hospital medical database, via follow-up visits and via the SHAR. We are aware of the risk that patients have been treated elsewhere; however, the multimodal search for complications ensured a low degree of loss to follow-up.

## Conclusions

In this retrospective multicenter cohort study of FNF patients, the cemented, polished, tapered Exeter stem was associated with a higher rate of PPF than the anatomic Lubinus SP2 stem. We suggest that an anatomic stem should be considered in the treatment of displaced FNF.

## Data Availability

The datasets used during the current study are not publicly available due to patient integrity, but are available from the corresponding author on reasonable request.

## Abbreviations

FNF: Femoral neck fracture
HA: Hemiarthroplasty
PPF: Periprosthetic fracture
SHAR: Swedish hip arthroplasty register
THA: Total hip arthroplasty
